# New 2D graphene hybrid composites as an effective base element of optical nanodevices

**DOI:** 10.3762/bjnano.9.125

**Published:** 2018-04-30

**Authors:** Olga E Glukhova, Igor S Nefedov, Alexander S Shalin, Мichael М Slepchenkov

**Affiliations:** 1 Department of Physics, Saratov State University, Astrakhanskaya street 83, 410012 Saratov, Russia; 2 Laboratory Nanooptomechanics, ITMO University, St. Petersburg, 197101, Russia; 3 Aalto University, School of Electrical Engineering, P.O. Box 13000, 00076 Aalto, Finland

**Keywords:** absorption coefficient, 2D CNT–graphene hybrid nanocomposite, optical conductivity, optical nanodevices, topological models

## Abstract

For the first time, we estimated perspectives for using a new 2D carbon nanotube (CNT)–graphene hybrid nanocomposite as a base element of a new generation o optical nanodevices. The 2D CNT–graphene hybrid nanocomposite was modelled by two graphene monolayers between which single-walled CNTs with different diameters were regularly arranged at different distances from each other. Spectra of the real and imaginary parts of the diagonal elements of the surface conductivity tensor for four topological models of the hybrid nanocomposite have been obtained. The absorption coefficient for p-polarized and s-polarized radiation was calculated for different topological models of the hybrid nanocomposite. It was found that the characteristic peaks with high intensity appear in the UV region at wavelengths from 150 to 350 nm (related to graphene) and in the optical range from 380 to 740 nm irrespective of the diameter of the tubes and the distance between them. For waves corresponding to the most intense peaks, the absorption coefficient as a function of the angle of incidence was calculated. It was shown that the optical properties of the hybrid nanocomposite were approximately equal for both metallic and semiconductor nanotubes.

## Findings

The applicability of graphene hybrid nanocomposites in the field of optical communications has been hinted to by the active research for the last six years, in which the unique properties of these hybrid nanocomposites as electro-optic materials for optical modulators of different types has been demonstrated [1–4]. One of the newest and hitherto only little investigated modifications of graphene is a 2D-hybrid composite composed of graphene monolayers and CNTs covalently bonded to them [5–8]. The hybrid 2D film exhibits high performance as photosensitive element of photodetectors in the range of 100–700 nm. It was found that a single photon absorbed by the film induces electron transport of 10^5^ electrons, and the response time amounts to ca. 100 microseconds [9]. It should be noted that modern synthesis technologies for such composites have allowed us to provide “cross-linking” between CNTs and graphene during synthesis without further scattering of charge carriers by defects [10–11].

The purpose of this work is the evaluation of perspectives for using the new 2D CNT–graphene hybrid nanocomposite as a base element of new optical nanodevices. Predictive in silico investigations were carried out using the popular and reliable quantum-mechanical SCC DFTB method [12–13]

**Figure 1:**
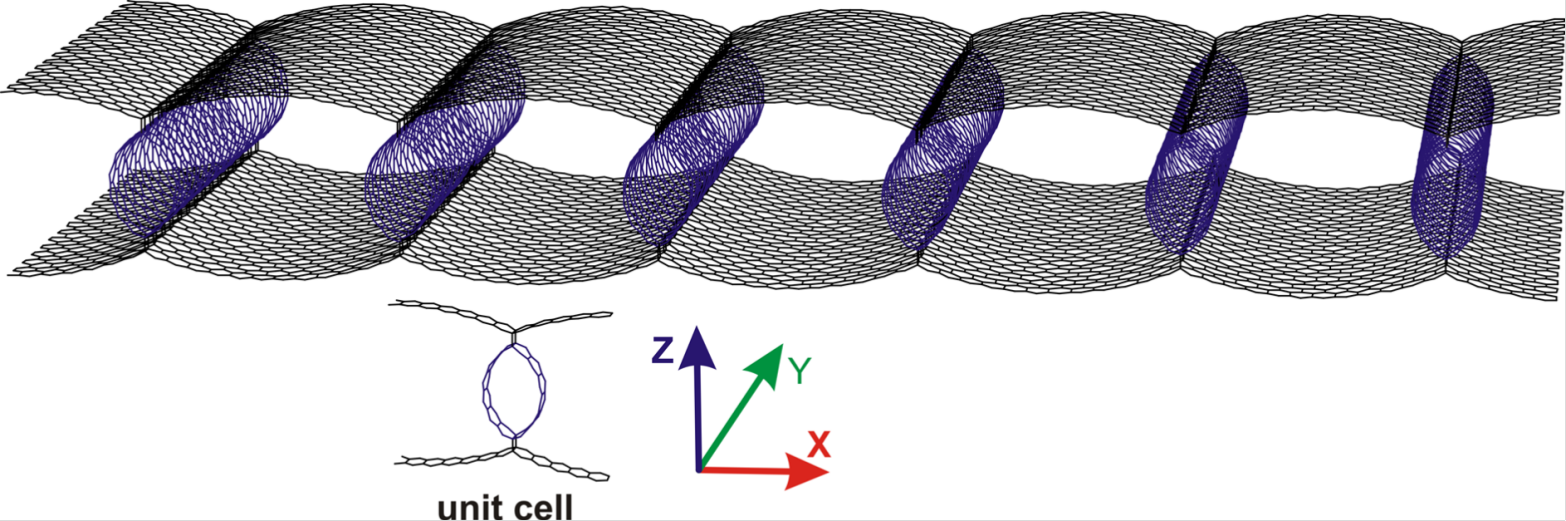
Topological model of 2D CNT-graphene hybrid nanocomposite.

The 2D CNT–graphene hybrid film was modelled by two graphene monolayers between which single-walled CNTs with different diameters were regularly arranged at different distances from each other. As was shown earlier [14], the composites with zigzag tubes (*n*, 0) (*n* = 10, 12, 14, 16, 18, 20) at a distance of 9–15 hexagons (with the step equal to unity) between them are thermodynamically stable. In this work, two composite models for the tubes (12,0) and (18,0) with metallic conductivity, and two models of semiconducting tubes (14,0) and (16,0) have been considered. These topological forms were previously discovered by experimental investigations [11]. The atomistic model of the composite unit cell was obtained by means of the original ”method of magnifying glass” described in detail in [14] using the SCC DFTB method. Figure 1 shows a general view of the composite fragment with its unit cell. Blue balls mark atoms of CNT, black balls mark atoms of graphene.

**Figure 2:**
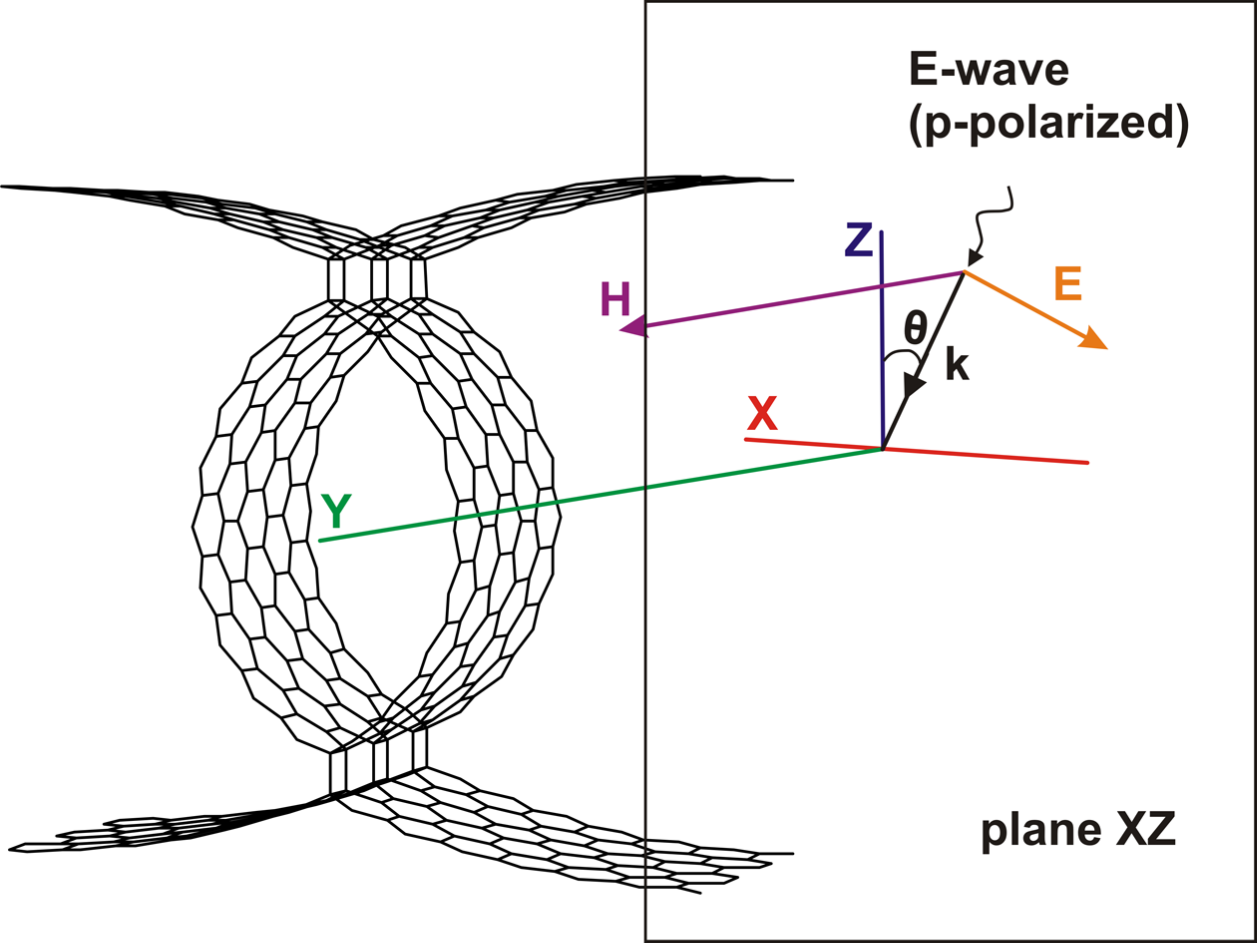
A fragment of the 2D CNT–graphene hybrid nanocomposite and configuration of an incident electromagnetic wave.

Investigation of the interaction with the incident electromagnetic waves (EMW) in the optical, UV, and IR ranges was performed based on Maxwell's equations. Figure 2 shows one of the configurations for the wave vector of the incident wave with respect to the atomic cell of the hybrid nanocomposite. In this case a plane electromagnetic wave with the wave vector *k* falls on the composite, which lies in the *XZ*-plane. The angle θ is the angle of incidence, the vectors **E** and **H** correspond to the electric field strength and magnetic field strength, respectively. The host medium is vacuum. In this configuration the wave is p-polarized (or E-wave).

To determine the coefficient of reflection, transmission and absorption, Maxwell's equations for the electric and magnetic fields in a vacuum with the 2D CNT–graphene composite as an interface have been considered. Assuming a plane-wave solution, Maxwell's equations can be written in the form

[1]
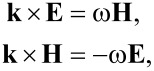


where **E** and **H** are the electric and the magnetic field strength, respectively, **k** is the wave vector and ω is the frequency of the incident electromagnetic radiation. The following boundary conditions were specified at the interface:

[2]
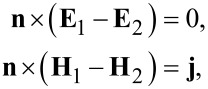


where **j** = σ*_xx_E_x_* + σ*_yy_E_y_* is the surface current density in the hybrid nanocomposite, induced by the incident radiation, σ*_xx_* and σ*_yy_* are the components of the 2D surface conductivity tensor. The indices 1 and 2 refer to the fields in the half-spaces *z* > 0 and *z* < 0, respectively, **n** is a normal vector to the surface. In future, we intend to solve the boundary-value problem for the two separate cases of the polarization of the incident radiation, either parallel to the plane of the incident radiation (p-polarization) or normal (s-polarization). A well-known scheme for obtaining the relations between the amplitudes of the incident, refracted and reflected s- and p-polarized waves when passing through the interface based on Maxwell's equations was used [15]. Assuming a value of the amplitude of the electric field equal to 1, one can write for the case of a p-polarized wave:

[3]
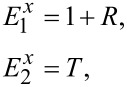


where *R* and *T* are the reflection and the transmission coefficient, respectively. Due to continuity of the tangent components of the electric field at the composite surface one can write:

[4]
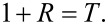


For the tangent components of the magnetic field at the composite surface one can write:

[5]
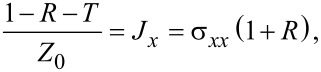


where *Z*_0_ is the characteristic impedance of free space and *J_x_* is the *x*-component of the surface current density vector. The final expression obtained for the reflection and transmission coefficients, *R* and *T*, in the case of a p-polarized incident wave takes the following form:

[6]
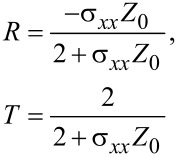


and in the case of s-polarization

[7]
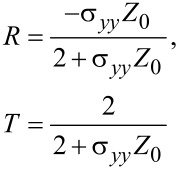


where *Z*_0_ is defined for the p-polarized wave as *Z*_0_ = *E_x_*/*H_y_* = η·cosθ, and for the s-polarized wave as *Z*_0_ = −*E_y_*/*H_y_* = η/cosθ, where θ is the angle of incidence η = 120π Ω is the input impedance of vacuum. Taking into account expressions for the reflection and transmission coefficients, is it possible to find the absorption coefficient by the following formula

[8]
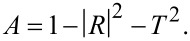


To calculate the elements of the complex optical conductivity tensor, the Kubo–Greenwood formula [16] that determines the conductivity as a function of photon energy Ω was used. It can be written as [17]:

[9]
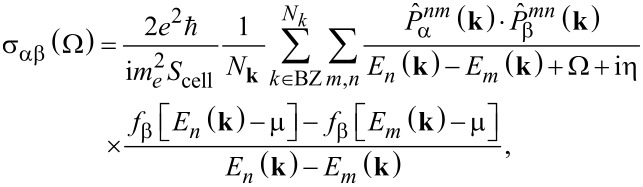


where *f*_β_(*x*) = 1/{1 + exp[β(*x* − μ)]} is the Fermi–Dirac function of the chemical potential μ with the inverse of the thermal energy β = 1/*k*_B_*T*; *S*_cell_ is the area of the supercell; *N***_k_** is the number of *k*-points needed to sample the Brillouin zone (BZ); 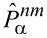 and 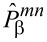 are the matrix elements corresponding to the α- and β-components of the momentum operator vector; *m_e_* and *e* are the free-electron mass and electron charge; *E_n_*(**k**) and *E_m_*(**k**) and are the sub-band energies of, respectively, valence band and conductivity band. The spin degeneracy is already taken into account in the above equations by the factor 2, η is a phenomenological parameter characterizing electron scattering processes.

**Figure 3:**
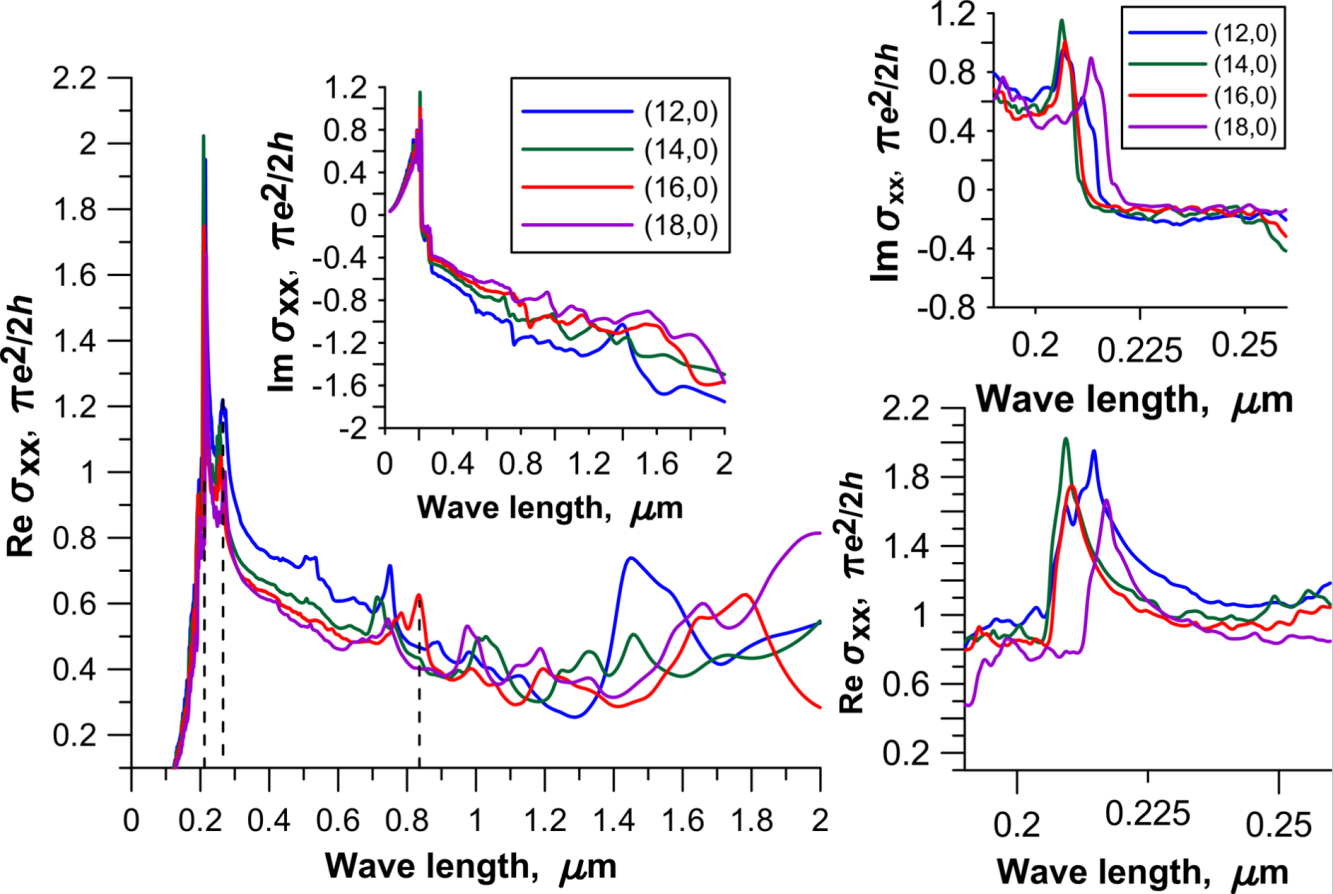
The optical conductivity of 2D CNT–graphene hybrid nanocomposites with an intertube distance of 13 hexagons in the direction perpendicular to the nanotube axis. The insets on the right side show the graphs of optical conductivity for the wavelength range of 190–260 nm.

**Figure 4:**
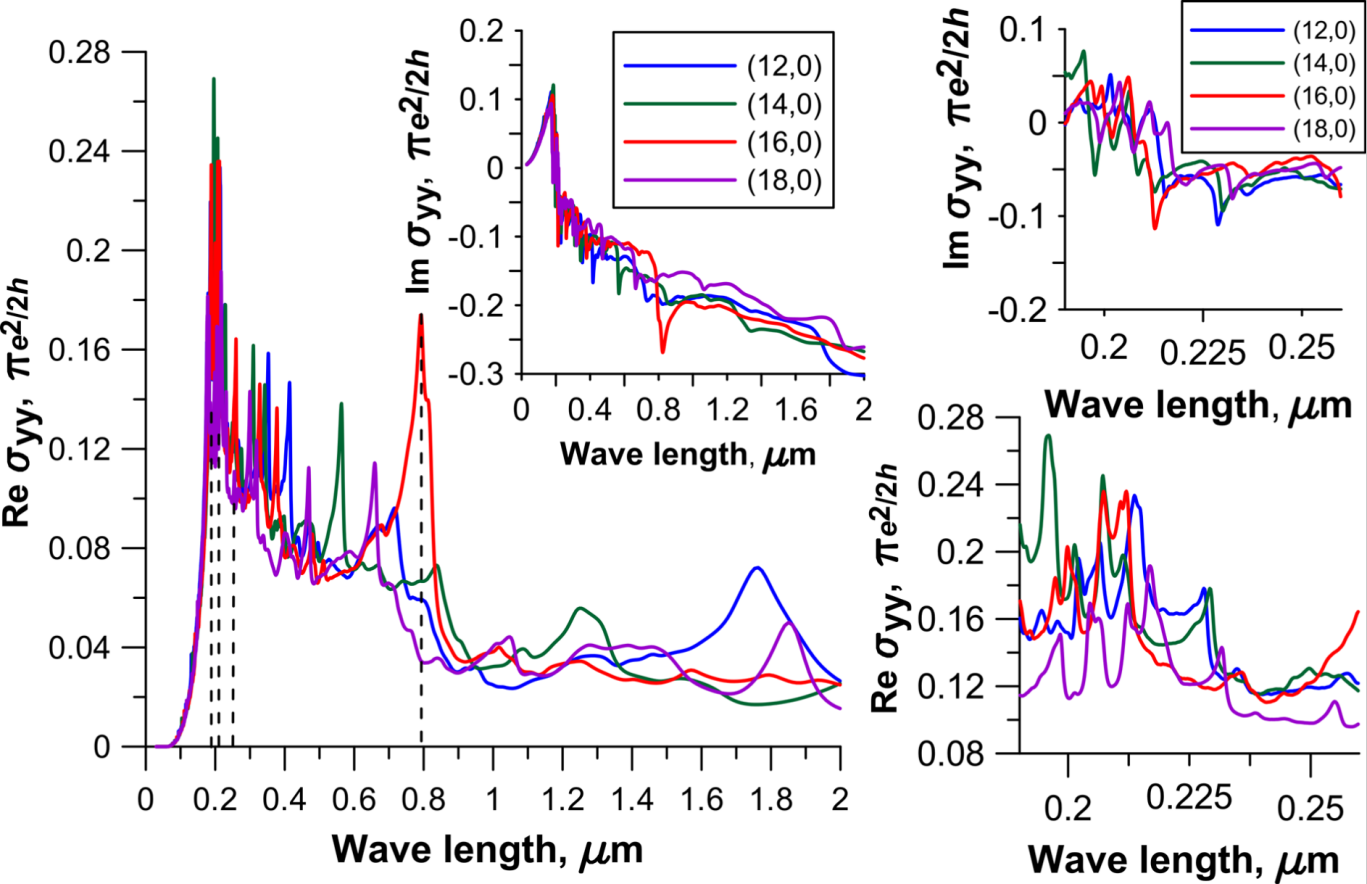
The optical conductivity of 2D CNT–graphene hybrid nanocomposites with an intertube distance of 13 hexagons along the nanotube axis. The insets on the right side show the graphs of optical conductivity for the wavelength range of 190–260 nm.

To calculate the elements of the impulse matrix 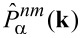, the known substitution *P*(**k**)→
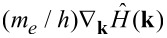
 was used, where 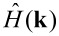 is the Hamiltonian. The detailed description for the calculation of the matrix elements of the momentum operator is given in [18]. The Hamiltonian was constructed within the SCC DFTB2 method. Figure 3 and Figure 4 show the spectra of the real and imaginary parts of the diagonal elements of the surface conductivity tensor for four topological models of 2D CNT–graphene hybrid nanocomposites with an intertube distance of 13 hexagons and four types of CNTs. An analysis of the spectrum profile for both tensor elements indicates the presence of prominent peaks in the wavelength range from 190 to 260 nm. The appearance of these peaks is due to the manifestation of pure graphene in the 2D CNT–graphene hybrid nanocomposite, so these peaks have a greater intensity for all the considered topological models hybrid nanocomposites. At the same time the spectrum profile of σ*_xx_* is similar to the spectrum of graphene, while the spectrum of σ*_yy_* has complex and multiple peaks. As previously shown [14], a complex profile of the conductivity spectrum along the nanotube axis is due to the influence of nanotubes. The appeared multiple peaks are characteristic for the conductivity spectrum of isolated individual CNTs. It should also be noted that the intensity of the maximum peak, observed at a frequency of 6 eV (206.6 nm) for pure free graphene, is reduced with the appearance of graphene ripple during the formation of the hybrid nanocomposite. As a result, the intensity of the peaks of the CNT–graphene film is higher than that of pure graphene and individual nanotubes (for details see Figure 6 in [14]).

Special attention should be paid to the peak of great intensity observed in the wavelength range of 800–830 nm for models with tube (16,0) (Figure 4). One can expect unusual properties of the hybrid nanocomposite when interacting with an incident electromagnetic wave in this range, in particular for reflected and absorbed waves. For other intertube distances the spectra are similar with only minor changes.

**Figure 5:**
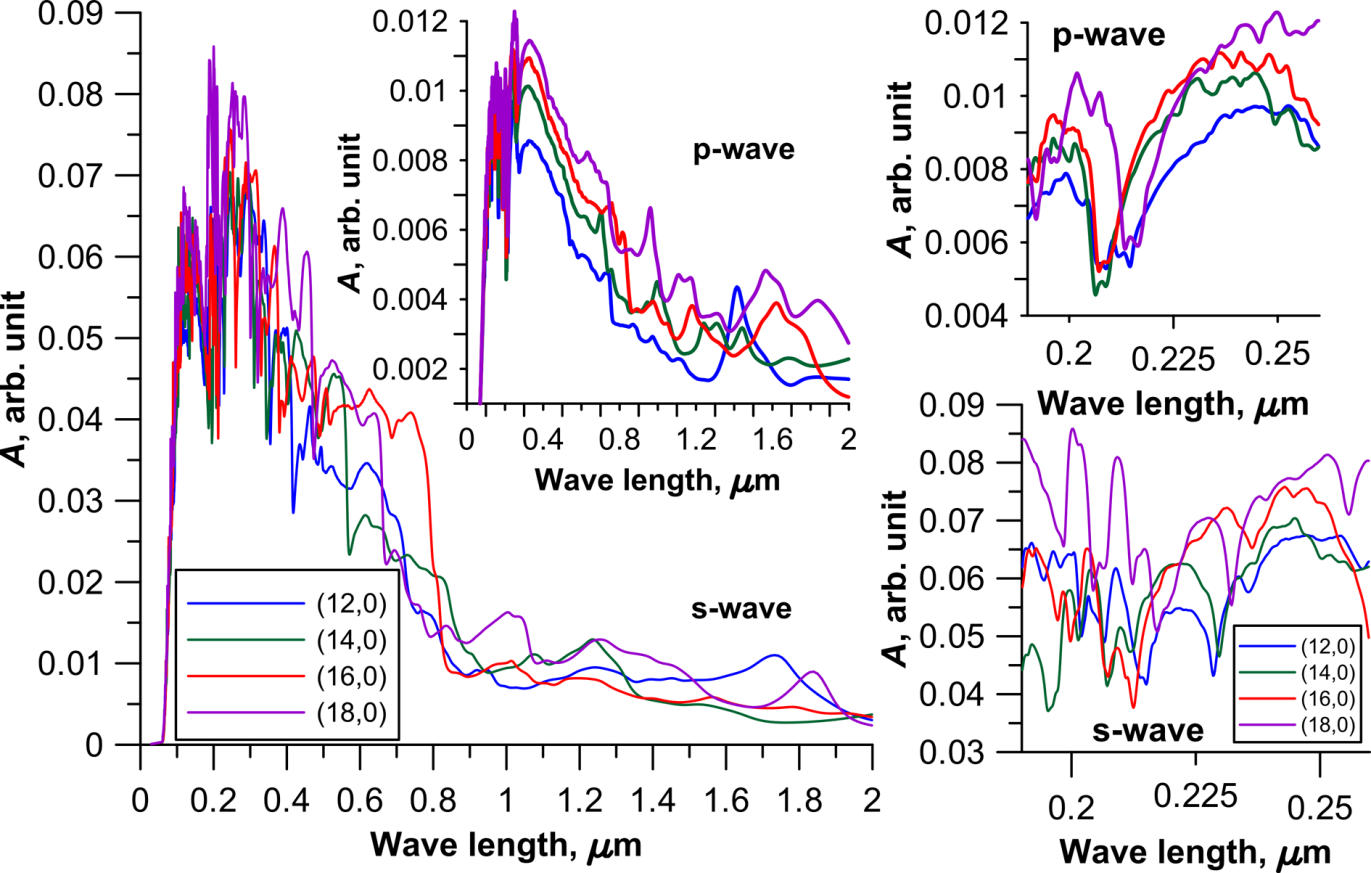
The absorption coefficient of 2D CNT–graphene hybrid nanocomposites with an intertube distance of 13 hexagons. The insets on the right side show the absorption coefficient for the wavelength range of 190–260 nm.

**Figure 6:**
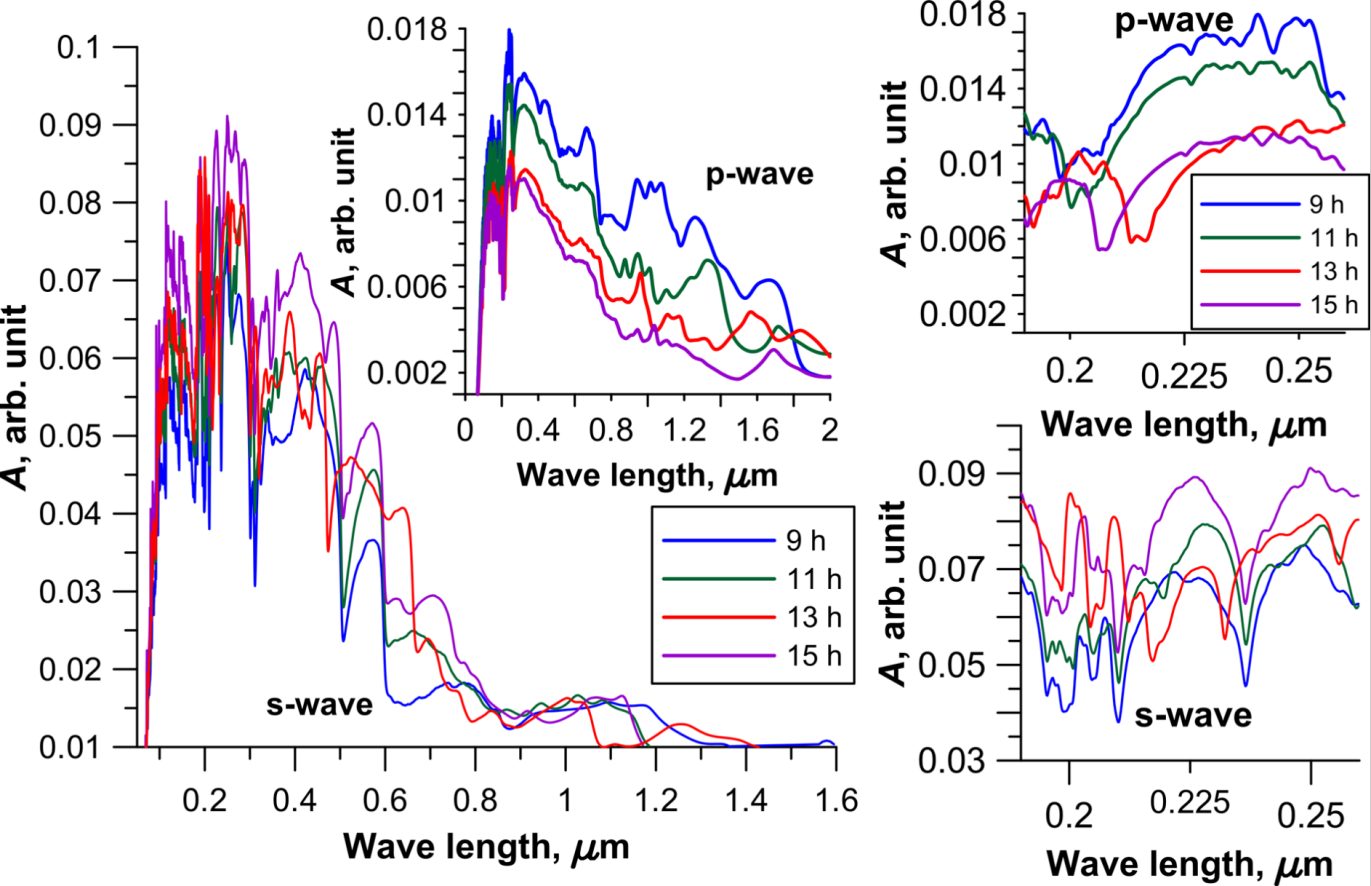
The absorption coefficient of 2D CNT-graphene hybrid nanocomposite with the same tube (18,0) and different intertube distances. The insets on the right side show the absorption coefficient for the wavelength range of 190–260 nm.

The calculation results of the absorption of electromagnetic waves of the CNT–graphene hybrid nanocomposite are presented in Figure 5 and Figure 6. These figures show two cases of the polarization for different topological models of the hybrid nanocomposite. Figure 5 shows the profile of the absorption coefficient (*A*) for four types of the tubes with an intertube distance of 13 hexagons, and Figure 6 presents the models of CNT–graphene hybrid nanocomposites with tube (18,0) at four intertube distances: 9, 11, 13 and 15 hexagons.

The analysis of the diagrams in Figure 5 and Figure 6, and also analysis of the calculated data for other models of the composite indicate characteristic peaks with high intensity for all topological models of CNT–graphene hybrid nanocomposites in the UV region at wavelengths from 150 to 350 nm (due to the graphene) and in the optical range from 380 to 740 nm. Intense peaks are absent in the IR region. The presence of the peaks with high intensity is typical for graphene at wavelengths from 150 to 250 nm, so the presence of peaks in the UV region is inevitable in this range. However, the maximum absorption of graphene is less than that of the CNT–graphene hybrid nanocomposite by almost 100%, i.e., the composite is more promising for the use in optical nanodevices than pure graphene.

**Figure 7:**
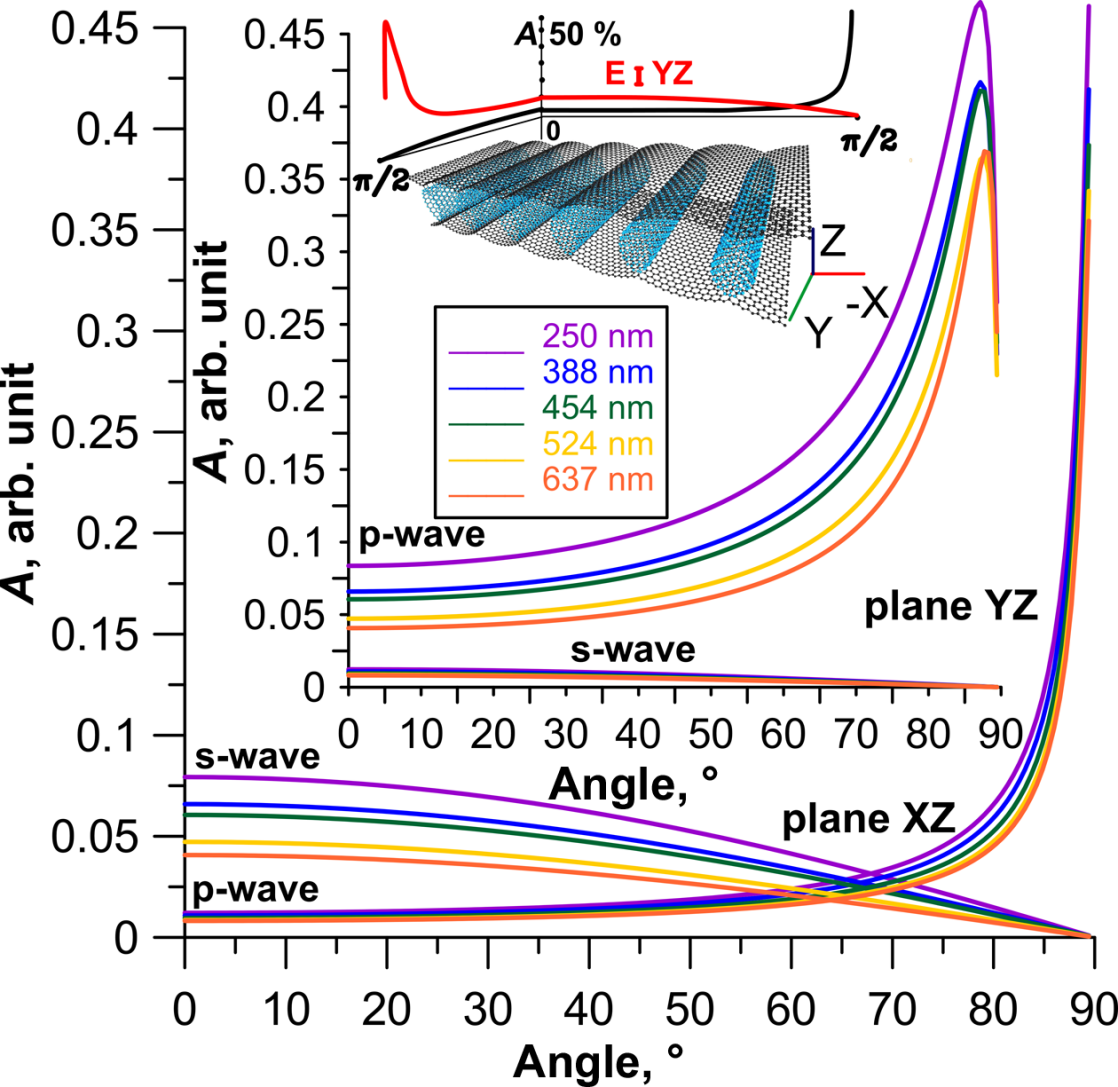
Absorption coefficient of a 2D CNT–graphene hybrid nanocomposite (tube (18,0), 13 hexagons intertube distance) as a function of the angle of incidence at different wavelengths.

For wavelengths corresponding to the most intense peaks, a diagram of the dependence of absorption coefficient (Equation 8) on the angle of incidence was calculated for two cases: 1) the wave vector lies in the *XZ*-plane; 2) the wave vector lies in the *YZ*-plane. Figure 7 shows the change in the absorption coefficient for two types of polarized waves incident at different angles on the film of tubes (18,0) between the graphene sheets at a distance of 13 hexagons from each other. Diagrams for wavelengths of 250, 388, 454, 524 and 637 nm were calculated. These values were chosen in accordance with the calculated graphs, similar to Figure 5 and Figure 6. This choice was due to perspectives for using the investigated CNT–graphene hybrid nanocomposite film as a working part of optical antennas or polarizers. According to Figure 7 for all wavelengths the maximum absorption is observed for a p-wave at incidence angles of 85–87° for the irradiation in the *YZ*-plane, and at angles of 85–90° for the irradiation in the *XZ*-plane. The absorption reaches values of 45–50% at these angles of incidence. Thus, one can say that the optical properties of the composite do not explicitly depend on the type of the tubes.

In summary, it can be concluded that the new 2D CNT–graphene hybrid nanocomposite is very promising for optoelectronic devices. In particular, the established regularities of change in absorbance as a function of the angle of incidence of the electromagnetic wave allows us to suggest the possibility of using the CNT–graphene film as a polarizer for electro-optical and magneto-optical thin film modulators. The advantages of such polarizers are a wide spectral range and low loss.
